# Systematic analysis of virus nucleic acid sensor DDX58 in malignant tumor

**DOI:** 10.3389/fmicb.2022.1085086

**Published:** 2022-12-19

**Authors:** Zhijian Huang, Limu Yi, Liangzi Jin, Jian Chen, Yuanyuan Han, Yan Zhang, Libin Shi

**Affiliations:** ^1^Department of Breast Surgical Oncology, Clinical Oncology School of Fujian Medical University, Fujian Cancer Hospital, Fuzhou, China; ^2^Department of Pathology, The First Affiliated Hospital of Guangdong University of Pharmacy, Guangzhou, China; ^3^Institute of Medical Biology, Chinese Academy of Medical Sciences and Peking Union Medical College, Kunming, China; ^4^Department of Pathology, Maternity and Child Healthcare Hospital of Longhua District, Shenzhen, China; ^5^Department of Nuclear Medicine, Clinical Oncology School of Fujian Medical University, Fujian Cancer Hospital, Fuzhou, China

**Keywords:** *DDX58*, pan-cancer, biomaker, immune infiltration, SARS-CoV-2

## Abstract

**Introduction:**

In December 2019, a novel epidemic of coronavirus pneumonia (COVID-19) was reported，and population-based studies had shown that cancer was a risk factor for death from COVID-19 infection. However, the molecular mechanism between COVID-19 and cancer remains indistinct. In this paper, we analyzed the nucleic acid sensor (*DDX58*) of SARS-CoV-2 virus, which is a significant gene related to virus infection. For purpose of clarifying the characteristics of *DDX58* expression in malignant tumors, this study began to systematically analyze the *DDX58* expression profile in the entire cancer type spectrum.

**Methods:**

Using TCGA pan-cancer database and related data resources, we analyzed the expression, survival analysis, methylation expression, mutation status, microsatellite instability (MSI), immune related microenvironment, gene related network, function and drug sensitivity of *DDX58*.

**Results:**

The expression level of *DDX58* mRNA in most cancers was higher than the expression level in normal tissues. Through TIMER algorithm mining, we found that *DDX58* expression was closely related to various levels of immune infiltration in pan-cancer. The promoter methylation level of *DDX58* was significantly increased in multiple cancers. In addition, abnormal expression of *DDX58* was related to MSI and TMB in multiple cancers, and the most common type of genomic mutation was “mutation.” In the protein–protein interaction (PPI) network, we found that type I interferon, phagocytosis, ubiquitinase, and tumor pathways were significantly enriched. Finally, according to the expression of *DDX58* indicated potential sensitive drugs such as Cediranib, VE−821, Itraconazole, JNJ−42756493, IWR−1, and Linsitinib.

**Discussion:**

In conclusion, we had gained new insights into how *DDX58* might contribute to tumor development, and *DDX58* could be used as an immune-related biomarker and as a potential immunotherapeutic target for COVID-19 infected cancer patients.

## 1. Introduction

Globally, 590 million cases of COVID-19 infection and 6.4 million deaths have been reported as of August 15, 2022. A recent study found that about 66 cancer patients were immunosuppressed with increasing susceptibility to infection and risk of serious complications (Al-Quteimat and Amer, 2020). Compared with other diseases, the genomes of 68 cancers have been fully studied. However, the gene information associated with COVID-19 remains largely unknown.

Genome-wide association study on COVID-19 patients with severe and critical illness showed that *DDX58* gene was closely associated to severe COVID-19. It is urgent to study the role of this gene in different cancers. RIG-I or DExD/H-box helicase 58 (*DDX58*) is a protein that recognizes viral double-stranded RNA and produces type I interferon, an antiviral and innate immune response medium as previous described (Morelli et al., 2021). At the same time, *DDX58* is considered as a potential novel target for COVID-19 treatment and a key component of COVID-19 infection and progress (Yamada et al., 2021). In cancer patients exposed to viruses, their condition worsened and their mortality increased (Han et al., 2021). Therefore, we aimed to find the role of *DDX58* in cancer immunotherapy, in order to provide a more suitable treatment idea for cancer patients infected with COVID-19.

Here, we showed the landscape analysis of *DDX58* expression level in healthy tissues and pan-cancer tissues using GTEx and TCGA, and then studied the relationship between *DDX58* and various tumor prognoses. We explored the relationship between *DDX58* and immune cell infiltration in specific tumor patients and studied the potential role of *DDX58* in tumor patients, we also analyzed the methylation profile of *DDX58* promoter and the mutation of *DDX58* in the UALCAN database. These findings might have important significance in preventing SARS CoV-2 infection and mitigate cytokine storm in patients infected with cancer. This study might also point out the therapeutic potential of *DDX58* inhibitors in preventing or mitigating SARS CoV-2 infection in specific cancer patients.

## 2. Materials and methods

### 2.1. Transcriptome data analysis

TCGA database and genotypic tissue expression (GTEx) database were used to obtain gene expression profiles. An analysis of 31 normal tissues was performed using mRNA data obtained from the GTEx project. Cancer cell lines were analyzed in 31 tissues according to their expression levels, and then the Kruskal Wallis test was performed on the mRNA data between adjacent tissues and tumor tissues, as well as healthy tissues and tumor tissues, to determine the difference of *DDX58* expression. *DDX58* expression levels were compared between healthy tissues and tumor tissues, as well as between adjacent tumor tissues and tumor tissues.

HPA^1^ contains normal tissue and tumor tissue protein levels of human gene expression profile information. In this study, we compared the expression of *DDX58* protein in normal tissues and cancer tissues of four different organs by HPA. The significance of the difference was calculated using the Wilcoxon test. *p* < 0.05 suggests that the expression of tumor tissue is different from that of normal tissue.

### 2.2. Clinical relevance analysis

The expression level of *DDX58* was examined using univariate COX regression analysis to determine whether it was associated with tumor prognosis in various cancers. According to the median of *DDX58*, samples were divided into two groups based on their expression levels: high-and low-expression groups of *DDX58*. In order to determine the importance of survival differences, a log rank test was used, with a threshold of *p* = 0.05. What’s more, we used the limma package to learn the relationship between *DDX58* and T stage in pan-cancer.

### 2.3. Construction and enrichment analysis of gene-gene, protein-protein and gene-disease networks

We constructed gene–gene interaction network through GeneMANIA^2^ and built PPI network through STRING database.^3^ We had further constructed a gene disease network on the OPENTARGET platform. Gene ontology (GO) terminology, Kyoto Encyclopedia of Genes and Genomes (KEGG) and GSEA were used to gene enrichment analysis. The term “GO” refers to molecular function (MF), cellular components (CC), and biological processes (BP). Use the “ClusterProfiler” package to perform GO, KEGG analysis, and GSEA. The TIMER^4^ was a comprehensive online database, analysis of a wide variety of cancer types related to immune infifiltrating. In this study, we used TIMER to determine the relationship between *DDX58* expression and ACE2.

### 2.4. Epigenetic methylation analysis and association analysis of methyltransferase

As a form of DNA chemical modification, DNA methylation controls gene expression by changing epigenetics without changing DNA sequence. To analyze the methylation level of tumor and normal tissues, we obtained them from the methylation module of UALCAN database. Later, from UCSC^5^ database, we have downloaded a standardized pan-cancer dataset: TCGA Pan Cancer (PANCAN, *N* = 10,535, G = 60,499), from which we further extracted the expression data of *DDX58* gene and 44 marker genes of three kinds of RNA modified m6A genes in each sample. We filtered the samples from: Primary Blood Derived Cancer-Peripheral Blood, Primary Tumor. Further, log2 (x + 1) transformation has been performed for each expression value. Next, we had calculated the spearman correlation between *DDX58* and marker genes of five different immune pathways.

### 2.5. Analysis of tumor mutation load and genome changes in pan-cancer

The total number of substitutions, insertions and deletions per megabase in the coding region of tumor gene exons was used to calculate the tumor mutation load (TMB). We got the expression data of *DDX58* gene in every sample from the previously downloaded datasets, combined with the previously screened samples. In addition, we also had download the Simple Nucleotide Variation dataset of level4 of all TCGA samples processed through MuTect2 software from GDC (https://portal.gdc.cancer.gov/; Beroukhim et al., 2010). To calculate the tumor mutation burden (TMB), we used the TMB function of the R software package maftools (version 2.8.01). Then we integrated the TMB and gene expression data of the samples. Finally, we obtained the expression data of 37 cancer species after removing those with fewer than three samples in a single cancer species. Through cBioPortal resources,^6^ we had analyzed the genetic changes of *DDX58* in the TCGA dataset (Reimer et al., 2021). The gene changes and mutation sites of *DDX58* were obtained in the “Oncoprint,” “Cancer Type Summary,” and “Mutations” sub modules.

### 2.6. Analysis of immune checkpoint genes and new immune antigens

Biological phenomena such as gene fusion, deletion mutation and point mutation are called new antigens encoded by mutated genes in tumor cells. We had calculated the binding affinity score of epitopes with 8–11 amino acids of a certain length and the epitopes with a score less than 500 nm were defined as new antigens. Then, we ranked the predicted new antigens according to antigenicity index value, affinity and mutation allele frequency. In each tumor sample, scannedo was used to count the new antigens and analyze the relationship between *DDX58* expression and new antigens. The immune checkpoint genes had been extracted and analyzed along with the *DDX58* expression to further investigate their relationship.

### 2.7. *DDX58* expression and microsatellite instability analysis

From UCSC (see Footnote 5) database we had downloaded a standardized pan-cancer dataset: TCGA Pan Cancer (PANCAN, *N* = 10,535, G = 60,499). Based on the previously extracted expression data and screened samples, we obtained MSI (Microsatellite instance) scores of each tumor from the previous study (Gounder et al., 2022). Next, the MSI and gene expression data of the samples were integrated.

### 2.8. Immune infiltration analysis

We screened the metastatic samples from the following sources: Primary Blood Derived Cancer - Peripheral Blood (TCGA-LAML), Primary Tumor, and TCGA-SKCM. The gene expression profiles of each tumor were extracted, mapped to GeneSymbol, and further analyzed using the Timer method of the R software package IOBR (version 0.99.9, https://www.ncbi.nlm.nih.gov/pmc/articles/PMC8283787/; Li et al., 2017). The B cell, T cell CD4, T cell CD8, Neutrophil, Macrophage and DC infiltration scores of each patient in each tumor were reevaluated according to gene expression.

### 2.9. Drug sensitivity of *DDX58* in pan-cancer

To study the drug sensitivity of pan-cancer patients to *DDX58*, the CallMinerTM database was used^7^ to get activity data and RNA seq expression profile of NCI-60 compounds. In order to analyze and select drugs approved by FDA or clinical trials, R packages “impute,” “limma,” “ggplot2,” and “ggpubr” were used for analysis.

## 3. Results

### 3.1. Differential *DDX58* expression analysis in pan-cancer tissues and normal tissues

**Figure 1:**
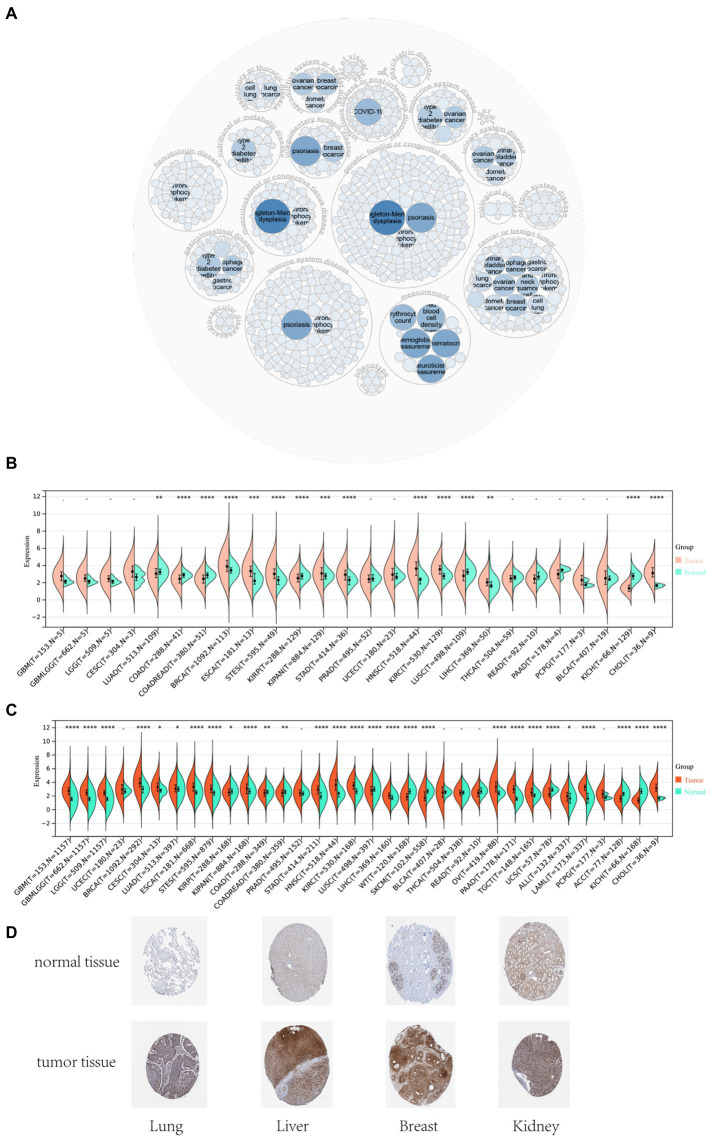
Differential expression analysis of *DDX58* in pan-cancer tissues and normal tissues. **(A)** *DDX58* related disease prediction **(B)** cancer and normal tissues in TCGA database **(C)** cancer and normal tissues in GTEx database **(D)** the protein expression level of *DDX58* in normal and tumor tissues of four different organs *p < 0.05, **p < 0.01, ***p < 0.001. BLCA, Bladder Urothelial Carcinoma; BRCA, Bladder Urothelial Carcinoma; CHOL, Cholangiocarcinoma, COAD, Colon adenocarcinoma; ESCA, Esophageal carcinoma, GBM, Glioblastoma multiforme; HNSC, Head and Neck squamous cell carcinoma; KICH, Kidney Chromophobe; KIRC, Kidney renal clear cell carcinoma; KIRP, Kidney renal papillary cell carcinoma; LIHC, Liver hepatocellular carcinoma; LUAD, Lung adenocarcinoma; LUSC, Lung squamous cell carcinoma; PRAD, Prostate adenocarcinoma; READ, Rectum adenocarcinoma; STAD, Stomach adenocarcinoma; THCA, Thyroid carcinoma; UCEC, Uterine Corpus Endometrial Carcinoma.

The analysis of gene disease network interaction showed that *DDX58* was mainly related to genetic, familial or genetic disease, immune system disease, infectious disease, benign tumor, etc. In particular, *DDX58* had a certain relationship with benign tumor (Figure 1A). Subsequently, we investigated the role of human *DDX58* expression in pan-cancer. A comparison of the expression levels of *DDX58* in tumors and normal tissues was performed using the TCGA database. As compared to normal tissues, *DDX58* was found to be highly expressed in BRCA, ESCA, STES, KIPAN, STAD, HNSC, KIRC, LIHC, CHOL, while it was low expressed in LUAD, COAD, READ, KIRP, LUSC, KICH (Figures 1B,C). At the same time, *DDX58* protein levels in four different organs and tissues also showed significant differences (Figure 1D).

### 3.2. Pan-cancer analysis of prognostic value of *DDX58* expression in different stages of cancers

**Figure 2:**
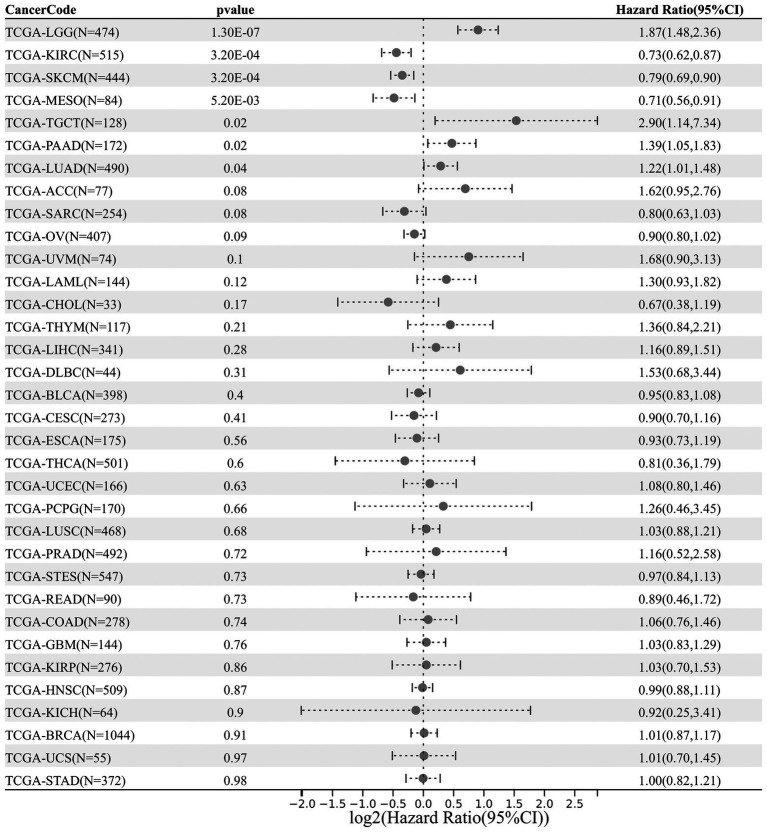
Pancancerous analysis of the diagnostic and prognostic value of *DDX58* expression. The forest map shows the HR and 95% CI of *DDX58* expression related to cancer OS.

Next, an analysis of *DDX58* expression and cancer prognosis was conducted using univariate Cox regression. According to the forest map of pan-cancer, the expression of *DDX58* had a significant impact on the OS of LGG, KIRC, SKCM, MESO, TGCT, PAAD, LUAD patients (Figure 2). In addition, we also analyzed the expression of *DDX58* in different cancer T stages, and the results showed that there were significant differences in the expression of *DDX58* in different stages of 10 cancers (Supplementary Figure S1).

### 3.3. Construction of *DDX58* gene, protein, disease network correlation with SARS CoV-2 receptor–ACE2

**Figure 3:**
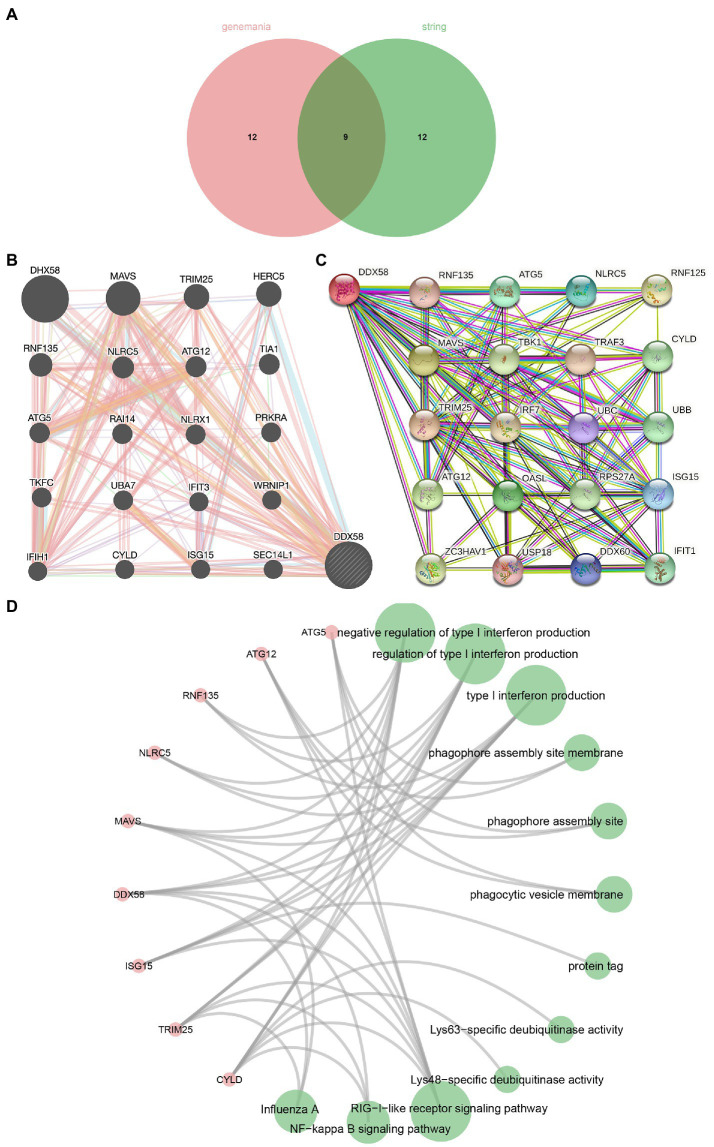
Gene, protein and disease networks use the *DDX58* related gene network mapped by GeneMANIA. **(A)** The Venn diagram where STRING and GeneMANIA intersect. **(B)** *DDX58* related gene network mapped by GeneMANIA **(C)** *DDX58* related protein network mapped by STRING. **(D)** Enrichment analysis of cross genes.

In order to understand the *DDX58* related network, the STRING and GeneMANIA protein-protein and gene-gene interaction networks that interact with *DDX58* were used (20 potential related genes were selected respectively; Figures 3B,C). We obtained 8 genes from the intersection of two data sets (Figure 3A) and carried out GO and KEGG analysis on 9 genes including *DDX58* (Figure 3D). We found that BP was enriched in negative regulation of type I interchange production, regulation of type I interchange production, type I interchange production. CC was mainly enriched in phagophore assembly site membrane, phagophore assembly site, phagocytic vascular membrane. MF was significantly enriched in protein tag, Lys63 specific dehydrogenase activity, and Lys48 specific dehydrogenase activity. KEGG analysis showed that many related pathways were significantly enriched, including RIG-I-like receiver signaling pathway, NF kappa B signaling pathway, Influenza A. In addition, it could be seen from the correlation analysis with AEC2 (SARS CoV-2 receptor) that there was a positive correlation between the expression of *DDX58* and ACE2 in many cancers (Supplementary Figure S2).

### 3.4. Epigenetic modification of *DDX58*

**Figure 4:**
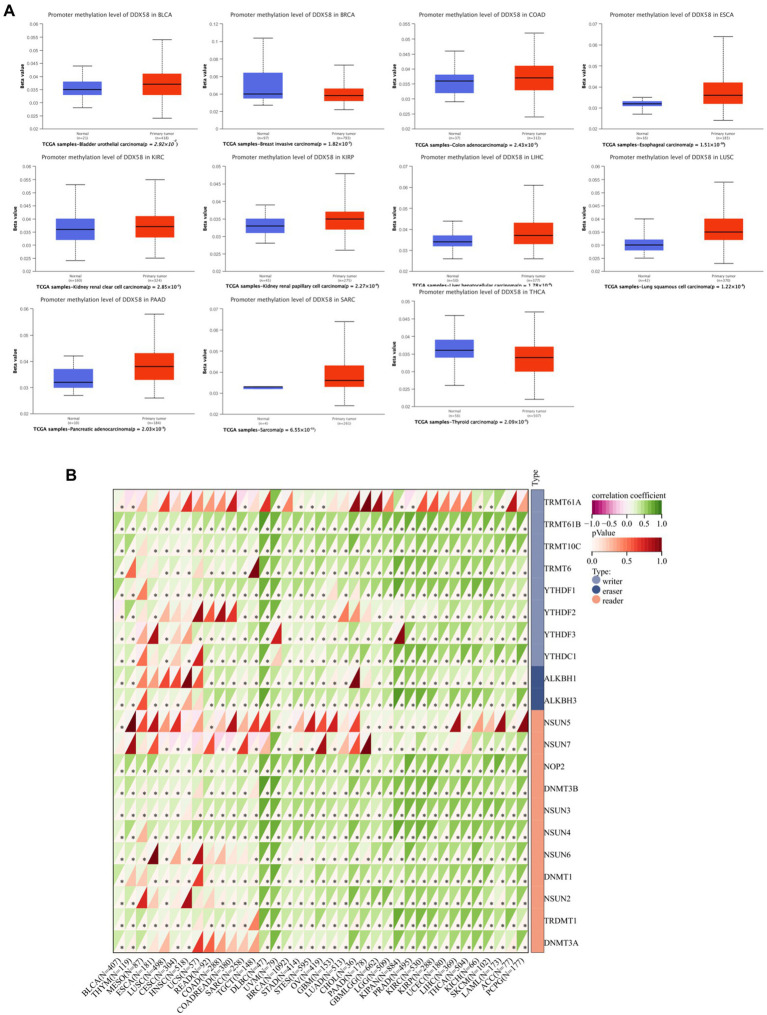
Correlation analysis between *DDX58* methylation level and methyltransferase expression level in pan-cancer tissues. **(A)** Display the difference of *DDX58* methylation level between tumor and adjacent normal tissues in TCGA database (β Value). **(B)** Correlation between *DDX58* expression and methylation related gene expression.

According to promoter methylation analysis, *DDX58* is hypermethylated in a variety of cancer types (Figure 4A). *DDX58* methylation seems to be correlated with the level of DNA methyltransferase mRNA expression in various cancers (all *p* < 0.05; Figure 4B). As we all know, DNA methylation is the result of DNA methyltransferase, which plays a role by covalently binding to the methyl at the 5′ carbon position of cytosine, a CpG dinucleotide in the genome. A correlation was found between methyl related genes and various cancers. There was a positive correlation between the expression of *DDX58* in pan-cancer and methyl-related genes, which meant that *DDX58* may mediate tumor genesis and progression by regulating epigenetic status. Moreover, it was worth noting that the correlation coefficient was higher in DLBC and UVM.

### 3.5. Genetic variation analysis of *DDX58* in pan carcinoma

**Figure 5:**
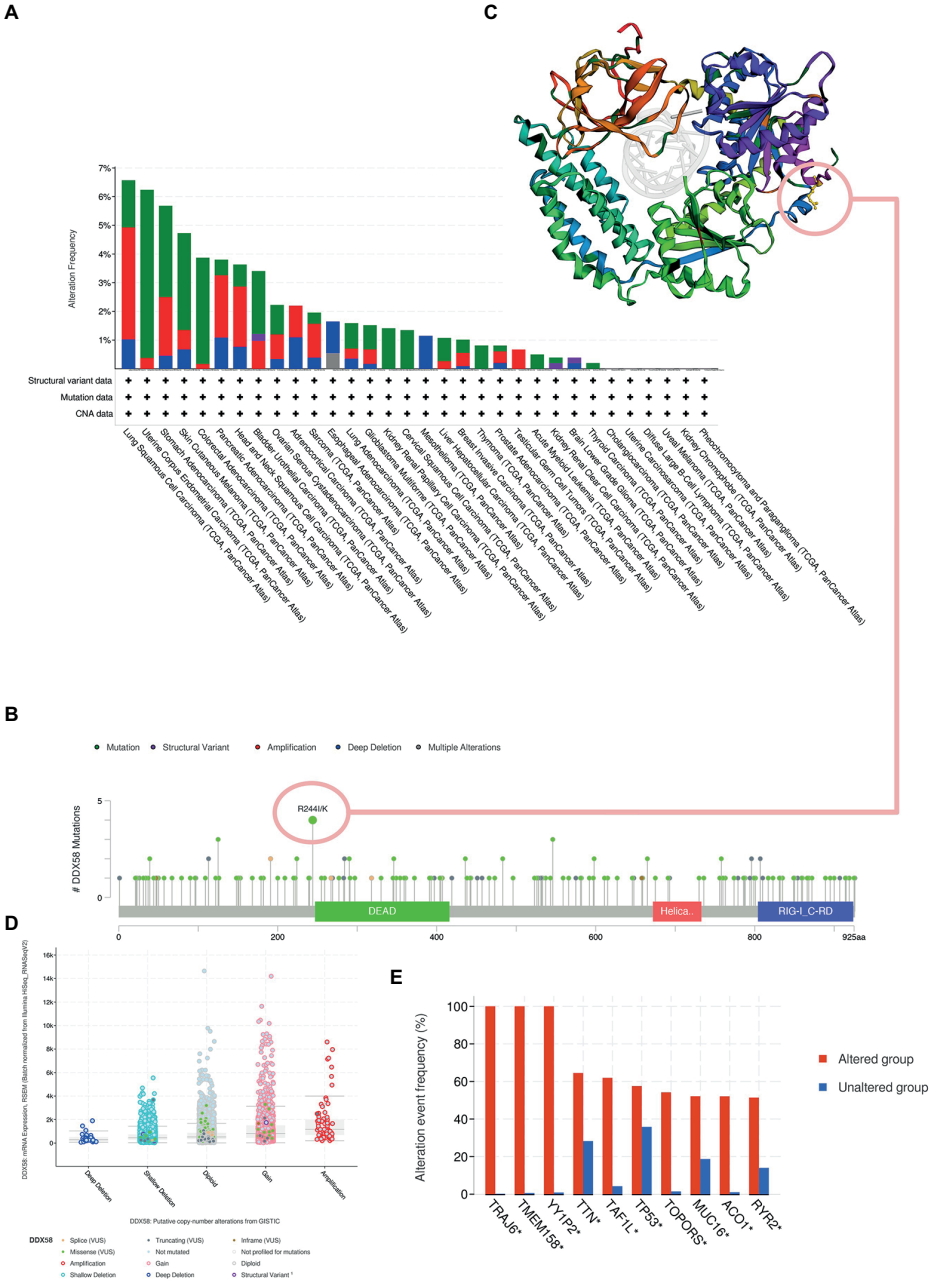
Genetic changes of *DDX58.* **(A)** Summary of *DDX58* changes in TCGA pan-cancer dataset. **(B)** The type, number and location of mutations in *DDX58* gene changes. **(C)** The 3D structure of *DDX58* at 232 mutation site. **(D)** The type of *DDX58* change in pan carcinoma. **(E)** Change frequency of related genes in *DDX58* changed and unchanged groups.

Based on the cBioPortal database, we found that there were higher *DDX58* gene changes in LUSC, UCEC, STAD, and SKCM, and mutation was the main type (Figure 5A). It further proved the type, location and quantity of *DDX58* gene modification. R244K/I changes were detected in 4 patients with *DDX58* (Figure 5B). Then the 3D structure of *DDX58* protein at this mutation site was mapped (Figure 5C). The most common type of mutation found in pan-cancer analysis were gain and diploid (Figure 5D). In addition, TRAJ6, TMEM158, YY1P2, TTN, TAF1L, TP53, TOPORS, MUC16, ACO1, RYR2 gene changes were more common in the altered group than in the unchanged group (Figure 5E).

### 3.6. *DDX58* is associated with TMB and MSI in some cancers

TMB and MSI are effective prognostic biomarkers and indicators of immunotherapeutic response in many tumors. From these two analyses, we can conclude the relationship between *DDX58* and immunotherapy prognosis of specific cancer types.

**Figure 6:**
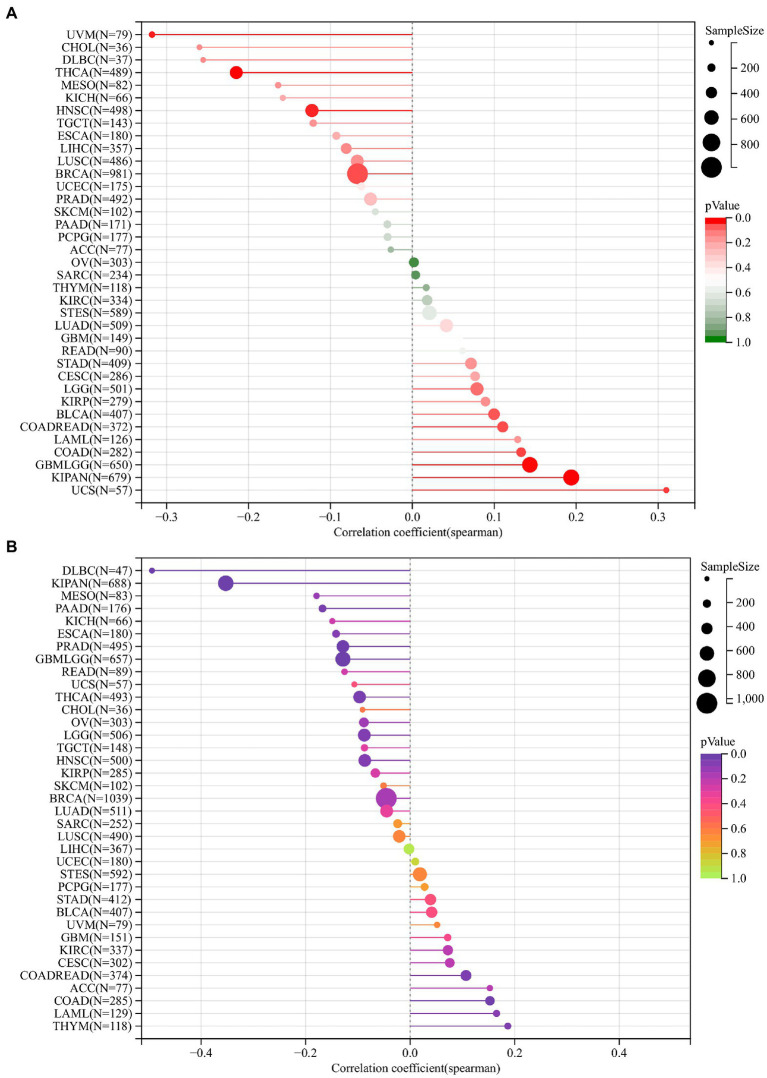
The relationship between the mRNA expression levels of TMB, MSI and *DDX58* in various cancers found in TCGA database. TMB was calculated based on the total incidence of mutations per megabase pair in each tumor, and MSI was calculated based on the total incidence of deletions or insertions in repeated sequences per megabase pair. **(A)** Correlation between TMB and DDX58 expression. **(B)** Correlation between MSI and *DDX58* expression. Spearman correlation test, *p* < 0.05 is significant.

The tumor cell genome’s TMB is usually expressed as the total number of non-synonymous mutations within an average 1 M base region. In some cases, it is also expressed directly as the number of somatic mutations. Base substitution, frameshift mutation, deletion mutation, insertion mutation and other mutation types are the most common mutation type. In tumor cells, TMB is a quantifiable indicator of mutation frequency. The correlation between *DDX58* and TMB was calculated for each tumor. Ten tumors showed a significant correlation, including a significant positive correlation in 6 tumors, such as GBMLGG (*N* = 650; R = 0.1430, *p* = 0.0002), COAD (*N* = 282; R = 0.1328, *p* = 0.0257), COADREAD (*N* = 372; R = 0.1103, *p* = 0.0334), KIPAN (*N* = 679; R = 0.194, *p* = 3.4926e-7), UCS (*N* = 57; R = 0.3099, *p* = 0.01895), BLCA (*N* = 407; R = 0.0996, *p* = 0.0444), significantly negative correlation in 4 tumors, for example: BRCA (*N* = 981; R = −0.0669, *p* = 0.0361), HNSC (*N* = 498; R = −0.1226, *p* = 0.0061), THCA (*N* = 489; R = −0.2149, *p* = 0.00001), UVM (*N* = 79; R = −0.3177, *p* = 0.0043; Figure 6A).

*DDX58* expression correlated with MSI in different types of cancer and we had calculated their spearman correlation in each tumor. A significant correlation was observed in 10 tumors, including significant positive correlation in 3 tumors, such as COAD (*N* = 285; R = 0.1526, *p* = 0.0098), COADREAD (*N* = 374; R = 0.1068, *p* = 0.0389), THYM (*N* = 118; R = 0.1868, *p* = 0.0428), and the significant negative correlation in 7 tumors, such as GBMLGG (*N* = 657; R = −0.1286, *p* = 0.0009) LGG (*N* = 506; R = −0.0878, *p* = 0.0483), KIPAN (*N* = 688; R = −0.3528,*p* = 1.351e-21), PRAD (*N* = 495; R = −0.1286, *p* = 0.0041), THCA (*N* = 493; R = −0.0967, *p* = 0.0317), PAAD (*N* = 176; R = −0.1677, *p* = 0.0260), DLBC (*N* = 47; R = −0.4937, *p* = 0.0004; Figure 6B).

It was worth noting that the absolute coefficients associated with TMB or MSI in the COAD cohort were relatively high compared with other cancer types, suggesting that the it may be sensitive to immunotherapy.

### 3.7. *DDX58* might regulate tumor immune microenvironment by influencing immune invasion of various cancer types and expression of immune checkpoints

**Figure 7:**
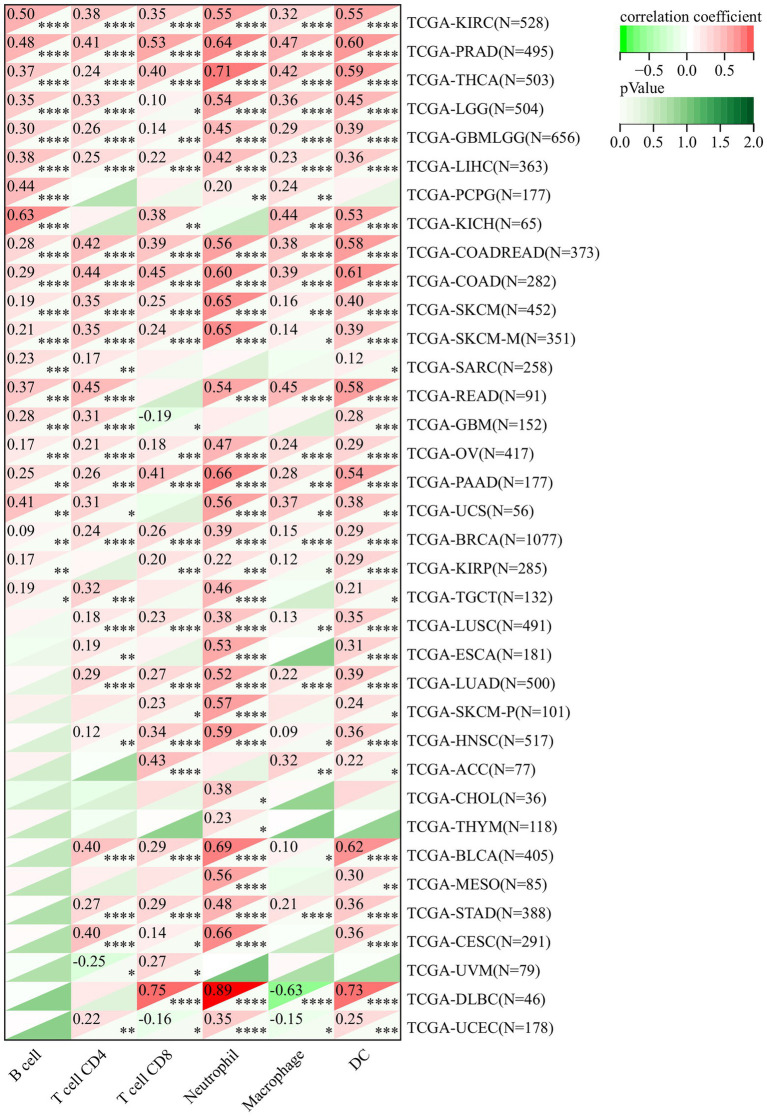
The expression level of *DDX58* mRNA calculated by TCGA and TIMER in the database was significantly correlated with the infiltration score of six common immune cells (B cells, CD4 + T cells, CD8 + T cells, neutrophils, macrophages, dendritic cells). Spearman correlation test, *p* < 0.05 is significant.

To determine whether this pathway affects the tumor immune microenvironment, we studied the expression of *DDX58* with the degree of immune cell infiltration in each cancer type. Using the data collected from TCGA and the six types of immune cells available in TIMER database (B cells, CD4 + T cells, CD8 + T cells, neutrophils, macrophages and dendritic cells) for analysis, the results indicated that there was significant correlation in multiple tumors (Figure 7). It was worth noting that CD8 + T cells had the highest DLBC correlation coefficient. Their corresponding linear regression diagram showed that the high expression of *DDX58* may be related to the increased level of immune cell infiltration. Similarly, *DDX58* also affected the expression of immune checkpoints in different cancers (Supplementary Figure S3).

### 3.8. *DDX58* drug sensitivity analysis

**Figure 8:**
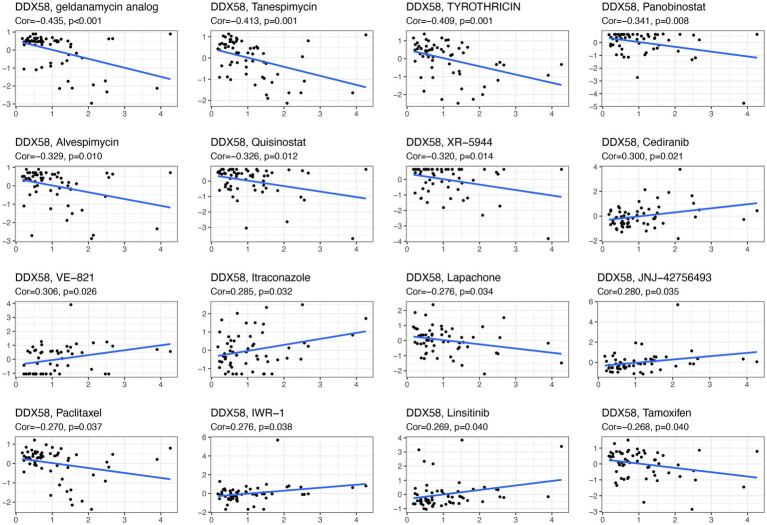
*DDX58* drug sensitivity analysis.

The database was further analyzed to determine whether *DDX58* expression was correlated with drugs using CellMiner™ (Figure 8). Our results indicated that the expression of *DDX58* was positively correlated with the sensitivity to Cediranib, VE−821, Itraconazole, JNJ−42756493, IWR−1, Linsitinib. And the expression of *DDX58* was negatively correlated with the drug sensitivity of geldanamycin analysis, Tanespimecin, TYROTHRICIN, Panobinostat, Alvespimycin, Quisinostat, XR−5944, Lapiphone, Paclitaxel,Tamoxifen.

## 4. Discussion

As of December 2019, COVID-19 had caused a worldwide pandemic and posed a serious threat to global public health (Talic et al., 2021). As a result of the COVID-19 pandemic, cancer patients were more likely to be infected with SARS CoV-2. According to these findings, COVID-19 might have an impact on cancer patients’ survival. RNA sensor RIG-I (*DDX58*) was a protein coding gene. The diseases related to RIG-I included Singleton Merten syndrome2 and Singleton Merten syndrome2. Signaling pathways that leaded to the production of type I interferon and proinflammatory cytokines in response to cytoplasmic viral nucleic acids (Bamming and Horvath, 2009; Shi et al., 2017; Zhao et al., 2017; Cadena et al., 2019). It formed ribonucleoprotein complex with viral RNA, on which homologous polymerization forms silk (Yoneyama et al., 2004; Sumpter et al., 2005). 3pRNA (RIG-1 agonist) treatment could increase cell death in melanoma cell lines and keep most melanoma cells in a non-proliferative state (Thier et al., 2022). In addition, RIG-1 activation inhibited STAT3/CSE pathway activity to restrain the proliferation of colon cancer cells (Deng et al., 2022).

Thus, to clarify how *DDX58* contributes to the pathogenicity of COVID-19, we must examine its relation to *DDX58*, this study systematically analyzed the expression profile of *DDX58* in the entire cancer type spectrum. Using TCGA pan-cancer database and related data resources, we analyzed the expression, survival analysis, methylation expression, mutation status, microsatellite instability (MSI), immune related microenvironment, gene related network, function and drug sensitivity of *DDX58*. Analysis of the relationship between *DDX58* expression and cancer immune invasion, tumor mutation, microenvironment and drug sensitivity had been finished, in order to determine *DDX58*’s potential for cancer immunotherapy and anti-COVID-19 treatment. We also carried out the correlation analysis between *DDX58* and AEC2 (SARS CoV-2 receptor; Supplementary Figure S2) to better understand the role of *DDX58* in COVID-19 and cancers.

In this study, we found the changes of *DDX58* mRNA in tumors. According to our research, pan-cancer was closely associated with the expression of *DDX58* protein. *DDX58* was highly expressed in BRCA, ESCA, STES, KIPAN, STAD, HNSC, KIRC, LIHC, CHOL, while it was low expressed in LUAD, COAD, READ, KIRP, LUSC, and KICH. *DDX58* was significantly associated with poor prognosis of LGG, TGCT, PAAD, LUAD, but significantly associated with improved prognosis of KIRC, SKCM, MESO patients. It indicated that *DDX58* might play different roles and functions in different cancers.

Eight genes were obtained by crossing the potential genes that interact with *DDX58* in the two databases, and nine genes including *DDX58* were analyzed by GO and KEGG. These 8 genes were ATG5, ATG12, RNF135, NLRC5, MAVS, ISG15, TRIM25, and CYLD, respectively. ATG5 usually combined with ATG12, catalyzed ATG7 and ATG10, played a role in autophagy, and regulates various functions of the body (Cui et al., 2022). It was known that RNF135 regulated the expression of IFN, and it participated in the RIG-I signal pathway by targeting RIG-I (Lai et al., 2019). NLRC5 could combine with LC3 to mediate MHC class I antigen presentation pathway (Zhan et al., 2022). MAVS mediated antiviral innate immunity (Zhang et al., 2022). The protein encoded by ISG15 gene was a ubiquitin like protein, when it was activated by interferon-αand-β, it binded to target proteins in cells. The encoded protein had a variety of functions, including chemotactic activity to neutrophils, orientation of junction target protein to intermediate filament, intercellular signal transduction and antiviral activity during viral infection (Jurczyszak et al., 2022). In responsd to ubiquitin E3 ligase and ISG15 E3 ligase (Zou and Zhang, 2006), TRIM25 played a role in the innate immune response to viruses by ubiquitinating *DDX58* and IFIH1 (Chiang et al., 2021). CYLD was a ubiquitin free enzyme that participates in NFκB activation and TNF-α induced necrosis (Dobson-Stone et al., 2020). Through enrichment analysis, it was found that these genes were associated with interferon related pathways, phagosomes, ubiquitination, RIG-I, NFκB related pathway, suggesting that it may affect the development of cancer through regulating immunity (Overman et al., 2017; Yang et al., 2021).

Disease network analysis found that *DDX58* was related to genetic, family or genetic disease, immune system disease, infectious disease, cancer or disease. This also showed that this gene was closely related to tumor and infectious diseases. Afterwards, we examined the relationship between *DDX58* expression and immune cell infiltration, and found that *DDX58* was significantly correlated with six types of immune cells (B cells, CD4 + T cells, CD8 + T cells, neutrophils, macrophages, and dendritic cells). In addition, abnormal DNA methylation was highly related to the occurrence, growth and carcinogenesis of tumors (Yang et al., 2021). Our study found that compared with their normal counterparts, cancer tissues were significantly hypermethylated, indicating that *DDX58* might promote tumor development by altering DNA methylation. However, the exact mechanism was still unclear. TMB and MSI are effective biomarkers to predict the prognosis of various tumors and indicators of immune response. TMB and MSI had been shown to be indicators of drug response in previous studies, particularly those that target immune checkpoint inhibitors such as CTLA4 and PD-1/PD-L1 (Overman et al., 2017; Mariathasan et al., 2018; Shim et al., 2020). Subsequently, we used the CellMinerTM database to find that the expression of *DDX58* was related to the sensitivity to many drugs, including Cediranib, VE−821, Itraconazole, JNJ−42,756,493, IWR−1, Linsitinib. These results are helpful to promote clinical drug guidance.

However, there were still some deficiencies in our research. First, based on bioinformatics analysis, there was a lack of relevant experimental or clinical data. In addition, although there was a correlation between the expression of *DDX58* in some tumors and survival rates, and *DDX58* changed the infiltration of immune cells, we were unable to establish a direct causal relationship. Future biological research needs to further clarify and confirm the role of *DDX58* in cancer.

In conclusion, the expression level of *DDX58* was significantly different in pan carcinoma.Turning RIG-I Sensor Activation Against Cancer had been used in clinical trails (Iurescia et al., 2020).And it had been proved that SARS CoV-2 M protein could inhibit the expression of IFNb and interferon stimulated genes induced by RIG-1(Sui et al., 2021). However, how *DDX58* played a role in these two diseases had not been reported. As an immune related biomarker, *DDX58* could be used to diagnose and predict the prognosis of COVID-19 cancer patients and their potential therapeutic targets.

## 5. Conclusion

We found that *DDX58* expression, survival prognosis, methylation, MSI, TMB, tumor immune microenvironment and drug sensitivity were different in pan-cancer. It was expected that *DDX58* might become a potential target for COVID-19 cancer therapy based on its abnormal expression in pan-cancer and significant differences in prognosis and immune environment. As a result, this study provided new insight into *DDX58*’s possible role in drug regulation as well as exploring its multiple roles in pan-cancer.

## Data availability statement

The original contributions presented in the study are included in the article/Supplementary material, further inquiries can be directed to the corresponding authors.

## Author contributions

LS, YZ, and YH conceived and designed the study. ZH, LY, and LJ performed the experiments. LY and JC analyzed the data. ZH and LY wrote the manuscript. All authors contributed to the article and approved the submitted version.

## Funding

This work was supported by Joint Funds for the innovation of science, Technology, Fujian province (Grant number: 2020Y9039) and Medical Research Fund of Guangdong (No. 2021112015285821).

## Conflict of interest

The authors declare that the research was conducted in the absence of any commercial or financial relationships that could be construed as a potential conflict of interest.

## Publisher’s note

All claims expressed in this article are solely those of the authors and do not necessarily represent those of their affiliated organizations, or those of the publisher, the editors and the reviewers. Any product that may be evaluated in this article, or claim that may be made by its manufacturer, is not guaranteed or endorsed by the publisher.

## Footnotes

^1^ https://proteinatlas.org/

^2^ https://genemania.org/

^3^ https://string-db.org/

^4^ https://cistrome.shinyapps.io/timer/

^5^ https://xenabrowser.net/

^6^ http://www.cbioportal.org/

^7^ https://discover.nci.nih.gov/cellminer
