# Supplementary figures and images for: Systematic analysis of virus nucleic acid sensor DDX58 in malignant tumor
Source: Front Microbiol. 2022 Dec 19;13:1085086. doi: 10.3389/fmicb.2022.1085086 (PMC9807228; doi:10.3389/fmicb.2022.1085086)

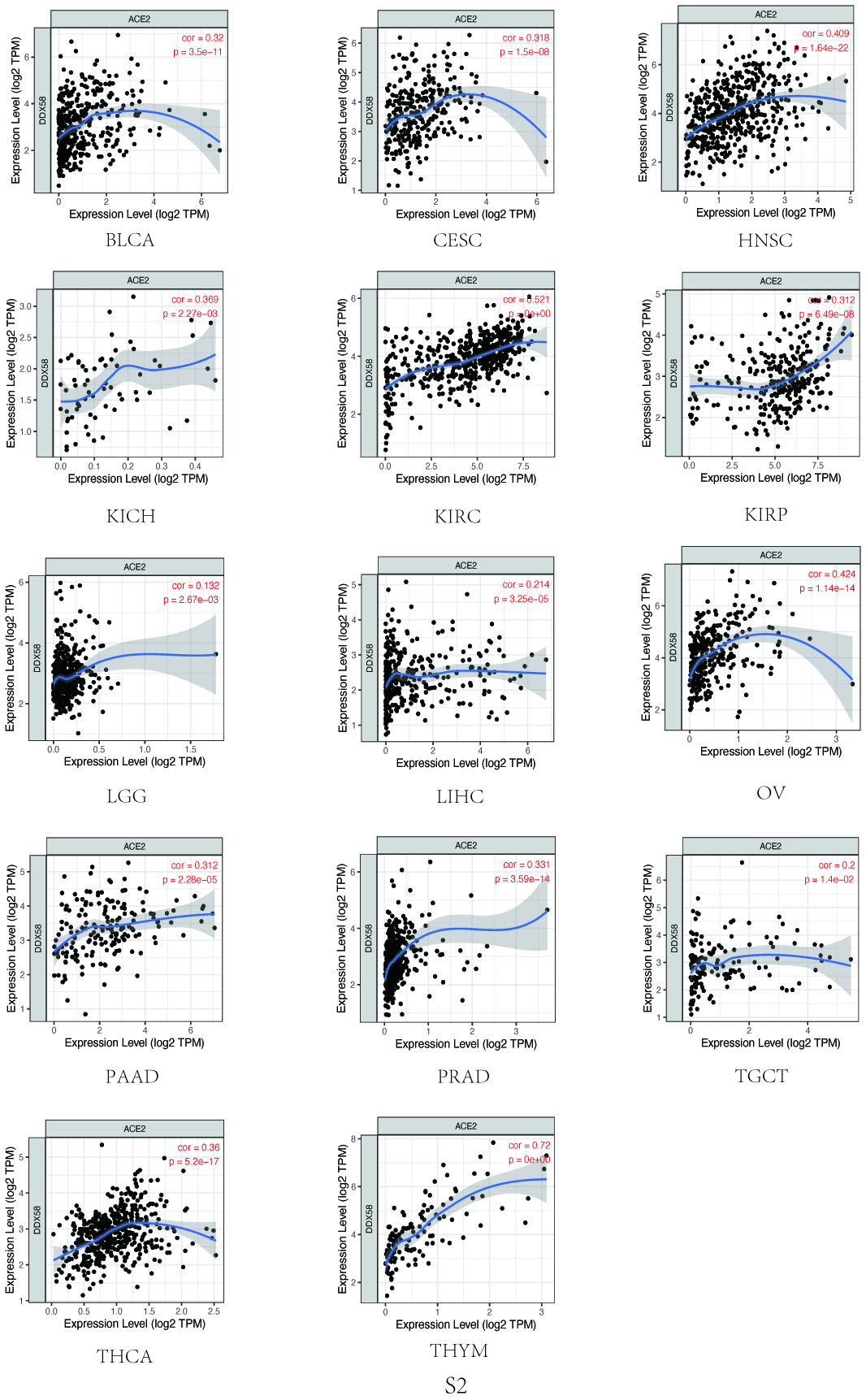

Supplement: Supplementary file 1 [file Data_Sheet_1.ZIP › Supplymentary Materials/ACE2.tif]

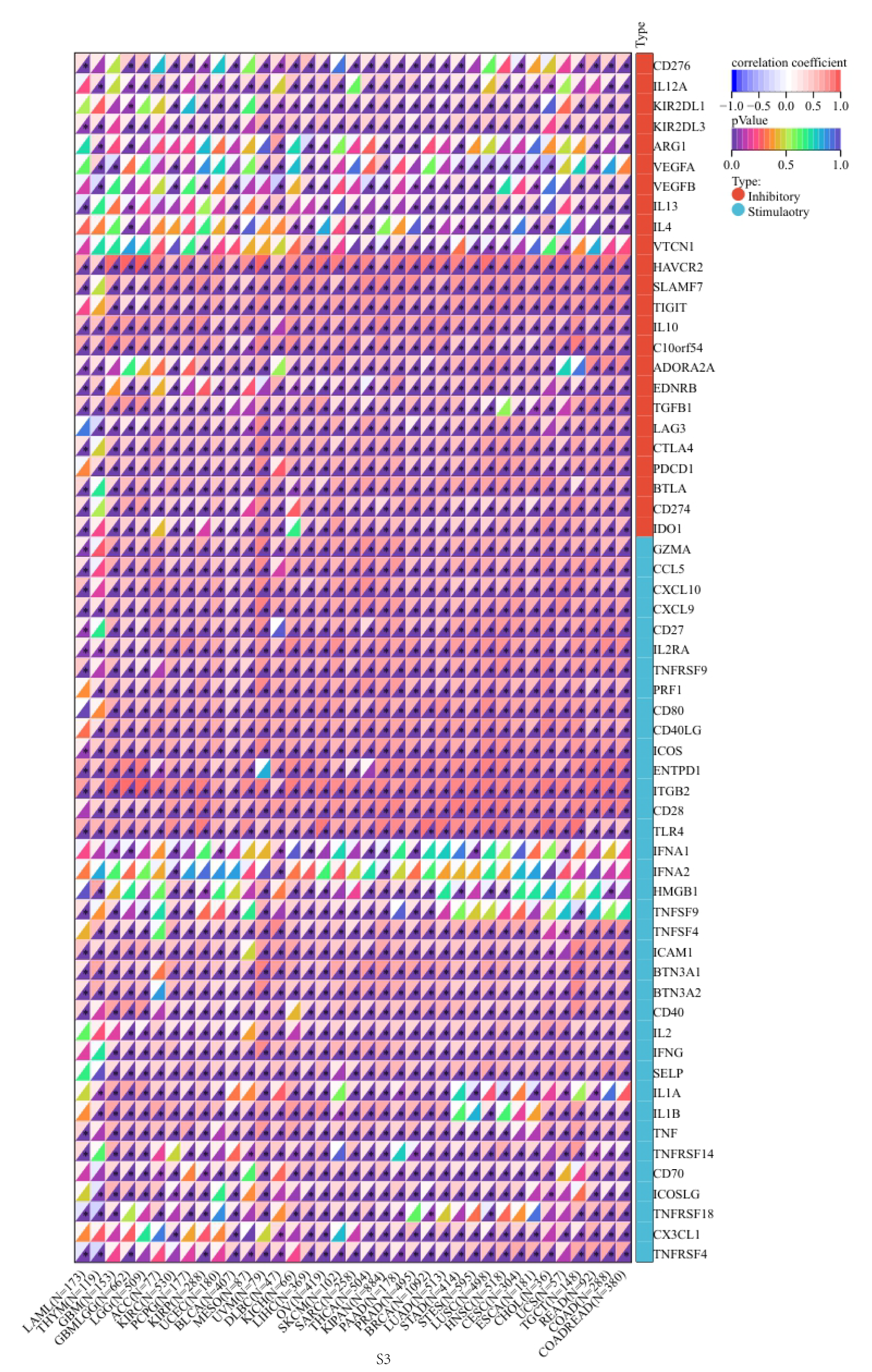

Supplement: Supplementary file 1 [file Data_Sheet_1.ZIP › Supplymentary Materials/σàìτû1⁄2μúÇμƒÑτé╣.tif]

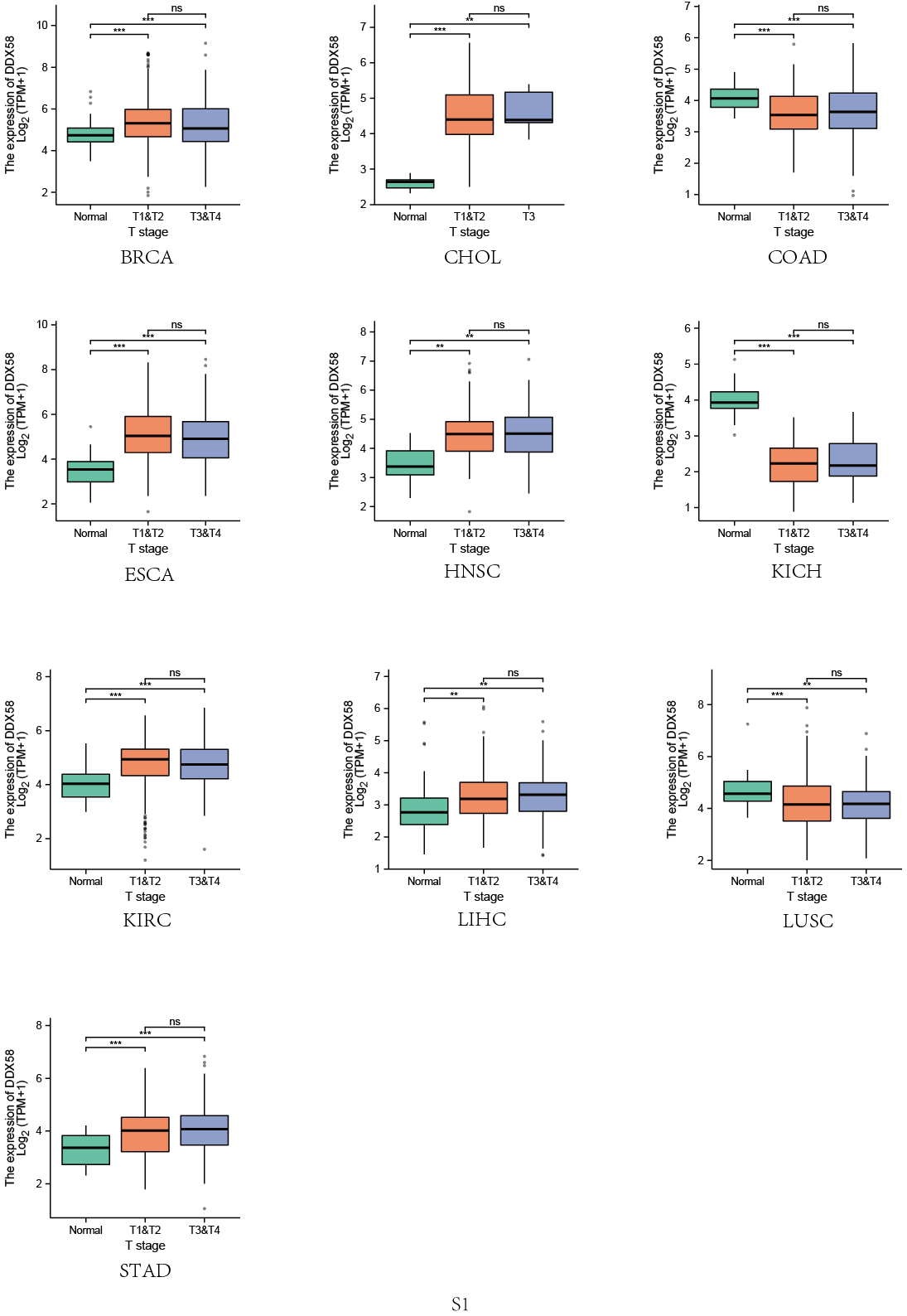

Supplement: Supplementary file 1 [file Data_Sheet_1.ZIP › Supplymentary Materials/Σ╕┤σ║èσêåμ£ƒ.tif]
